# Handover Control for Human-Robot and Robot-Robot Collaboration

**DOI:** 10.3389/frobt.2021.672995

**Published:** 2021-05-07

**Authors:** Marco Costanzo, Giuseppe De Maria, Ciro Natale

**Affiliations:** Dipartimento di Ingegneria, Università degli Studi della Campania Luigi Vanvitelli, Aversa, Italy

**Keywords:** object handover, tactile sensing, robotic manipulation, human-robot collaboration, cooperative robots

## Abstract

Modern scenarios in robotics involve human-robot collaboration or robot-robot cooperation in unstructured environments. In human-robot collaboration, the objective is to relieve humans from repetitive and wearing tasks. This is the case of a retail store, where the robot could help a clerk to refill a shelf or an elderly customer to pick an item from an uncomfortable location. In robot-robot cooperation, automated logistics scenarios, such as warehouses, distribution centers and supermarkets, often require repetitive and sequential pick and place tasks that can be executed more efficiently by exchanging objects between robots, provided that they are endowed with object handover ability. Use of a robot for passing objects is justified only if the handover operation is sufficiently intuitive for the involved humans, fluid and natural, with a speed comparable to that typical of a human-human object exchange. The approach proposed in this paper strongly relies on visual and haptic perception combined with suitable algorithms for controlling both robot motion, to allow the robot to adapt to human behavior, and grip force, to ensure a safe handover. The control strategy combines model-based reactive control methods with an event-driven state machine encoding a human-inspired behavior during a handover task, which involves both linear and torsional loads, without requiring explicit learning from human demonstration. Experiments in a supermarket-like environment with humans and robots communicating only through haptic cues demonstrate the relevance of force/tactile feedback in accomplishing handover operations in a collaborative task.

## 1 Introduction

Recent studies testify an increasing use of robots in retail environments with a number of objectives: to relieve staff from repetitive tasks with low added value; to reallocate clerks to customer-facing activities; to gather in-store data for real-time inventory to reduce out-of-stock losses (Retail Analytics Council, 2020). Commercial solutions already exist for automated inventory management, e.g., the Bossa Nova 2020, Tally 3.0 by Simberobotics, LoweBot by Fellow Robots or Stockbot by PAL robotics (Bogue, 2019). However, other in-store logistic processes, like product pre-sorting and shelf re-stocking are still difficult to automate, even though there is a strong interest from retailers, due to their high costs (Kuhn and Sternbeck, 2013). The most time consuming task is the shelf replenishment and 50% of such time is devoted to find the correct slot on the shelf. Only few scientific contributions exist on this logistic automation problem (Ricardez et al., 2020; Sakai et al., 2020), mainly related to the Future Convenience Store robotic challenge launched by the World Robotic Summit^1^. This is because the re-stocking task involves complex manipulation operations that are still a challenge for a robot. Dexterous manipulation of objects with unknown physical properties is very difficult, especially with simple grippers like parallel jaws, that are the most widespread devices for material handling. Adopting a fixed grasp with a parallel gripper can be very limiting when objects have to be handled in cluttered and narrow spaces. Changing the grasp configuration without re-grasping the object requires the ability to re-orient the object in hand, that is a pivoting motion about the grasping axis of the parallel gripper. Recent papers demonstrated that such in-hand manipulation abilities allow a robot to autonomously refill a supermarket shelf (Costanzo et al., 2020b; Costanzo et al., 2020c; Costanzo et al., 2021). Nevertheless, the whole re-stocking process requires execution of additional tasks that cannot be executed by robots, like opening cartons, removing packaging of multiple items before placing them on the shelf, or handling exceptions, like liquid spills. All these operations demand for complex cognitive and/or manipulative skills that are still a human prerogative. Therefore, with the current technology a step toward automation of retail logistics can be taken through execution of such processes in a collaborative way, where robots and humans are able to exchange objects, the so-called handover operation. Availability of a robot able to receive objects from the human clerk (H2R operation) can significantly alleviate the human workload since the robot can easily reach lower or higher shelf layers, that are outside the ergonomic golden zone. The dual robot-to-human operation (R2H) can be very useful in stores to help impaired or elderly people to retrieve products from the shelves. Analogously, in fulfilment centers of large on-line stores, robots can retrieve products from shelves and pass them to a human operator who takes care of their packaging. In a more futuristic scenario where robots can fully replace human clerks, the robot-to-robot (R2R) handover operation can be envisaged during the whole in-store logistic process.

The handover task is commonly defined in the robotics community as a joint action between two agents, the giver and the receiver (Ortenzi et al., 2020). It is usually divided into two phases, the pre-handover and the physical handover. In each phase, a number of aspects should be considered. For a detailed review of each aspect, the reader is referred to the survey by Ortenzi et al. (2020); in the following only a short review is presented with the aim to frame the contribution of the present paper within the literature.

**FIGURE 1:**
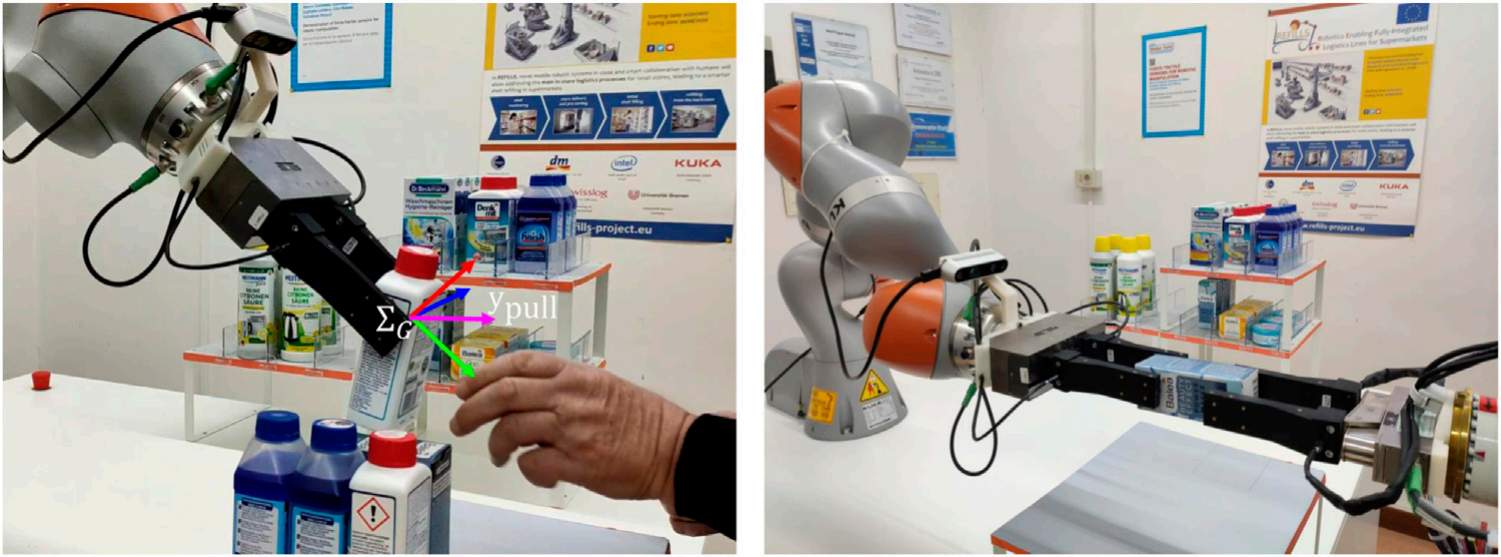
**Left picture:** Experimental setup for the robot-to-human and human-to-robot handover, the frame $\sum_{G}$ (in RGB convention) is the grasp frame, the magenta arrow represents the pulling direction $y_{\text{pull}}$ (defined in Section 3); **Right picture:** Experimental setup for the robot-to-robot handover. The robot on the left is the receiver and is equipped with an eye-in-hand camera to track the object to take. Both robots are endowed with force/tactile sensors.

The communication mechanism is crucial for initiating the action and to coordinate it as soon as it has started (Strabala et al., 2013). We address only the communication problem during the physical handover phase, and we adopt haptic cues as the sole communication mean between the two agents, even in the R2R handover the robot controllers do not share any further communication channel. The cues are a simple code based on the interaction force perceived by the agents through tactile sensing at fingertips. The code assumes that robots are able to measure all components of the force vector, thus the grippers are equipped with force/tactile sensors built in our laboratory (see the experimental setup in Figure 1).

Grasp planning is another fundamental problem to tackle for an effective handover execution. Ideally, the giver should grasp the object in such a way the receiver does not need to perform complex manipulation actions after the handover before using the object, as discussed, e.g., in Aleotti et al. (2014). In a R2H context, the robot should be capable of dexterous operations to hand the object over in a proper pose. Even parallel grippers are able to perform in-hand manipulation actions as demonstrated by Costanzo et al. (2020a). Both object and gripper pivoting maneuvers are used in this paper to achieve this objective, for instance, choosing an object orientation so as to avoid high torsional loads at receiver fingertips in case a precision grasp is needed to take the object. This can likely happen in a collaborative packaging task where the human operator has to arrange in a box the items passed by the robot. In the H2R context, as proved by many studies on human behavior, e.g. (Sartori et al., 2011), the giver grasps the object to hand over by considering the final goal of the receiver. If a desired orientation is required, the robot should apply a grasp force suitable to hold also the torsional load. To the best of our knowledge no other research deals with handover of objects subject to torsional load.

Still in the H2R operation, visual perception of the object location by the robot is challenging since it has to be performed with the object already grasped by the human, hence it is partially occluded. We adopt a texture-based approach that tracks 3D key points on the object surface detected by an eye-in-hand RGB-D camera. Hence, the robot directly tracks the object rather than the human hand, and no additional markers are needed, differently from the marker-based solutions proposed by Medina et al. (2016) and by Pan et al. (2018). Among the marker-less approaches, recent works are (Nemlekar et al., 2019; Yang et al., 2020; Rosenberger et al., 2021). Each of these works on H2R handover, and our paper, use object and/or human tracking with a different aim. Nemlekar et al. (2019) focus on reaching the handover location by tracking the human skeleton and predicting the so-called Object Transfer Point, Yang et al. (2020) focus on the choice of the grasp orientation based on the human grasp type, while Rosenberger et al. (2021) focus on the choice of a grasp that ensures the safety of the human using eye-in-hand vision. Our approach, instead, aims at reaching a grasp selected beforehand based on the task that the robot has to perform after the handover, i.e. placing the object on the shelf possibly using in-hand manipulation which requires specific constraints on the grasp. Differently from the solution by Yang et al. (2020), in our work the grasp motion is not planned but directly executed in a closed-loop fashion, this allows the robot to achieve a speed comparable to the human one while automatically reacting to the giver motion. The approach proposed by Rosenberger et al. (2021) is computationally demanding and it is still not suitable for real-time closed-loop control. In contrast, our approach requires an object database containing the grasp poses (this is a fair assumption in a supermarket scenario) but enables a fast closed-loop control and can track the object even if the human changes the object pose during the handover execution. The main limit of the solution by Nemlekar et al. (2019) is the need to keep the entire skeleton of the human body within the field of view of the camera to allow the tracking algorithm to work properly; this limit has been overcome by Rosenberger et al. (2021) who can track only parts of the human body as well as our approach which tracks the object only.

A fast visual servoing loop is used in this paper to control the robot receiver motion in such a way the handover is fluid, that is a primary requirement as discussed by Medina et al. (2016). This way, during the H2R operation the handover location is chosen by the giver and the receiver moves toward it in real-time. For instance, this is helpful if the human has a limited workspace and the robot has to reach an handover location compliant with the human constraints. When the robot approaches the object, the initial error between the actual and desired robot location could be high, in the classical algorithm by Marchand et al. (2005) this issue limits the dynamic performance of the visual servoing controller. This paper improves the dynamic performance of the visual controller in terms of speed by using a time-varying reference on the feature space to have a low tracking error and higher control gains.

During the physical handover phase the object load is shared by the giver and the receiver. A number of studies on the forces exchanged by humans during this phase have been carried out. For instance, Mason and MacKenzie (2005) found that the grip force of both giver and receiver is modulated during the object exchange. The giver decreases its grip force, while the receiver increases it until the load is transferred. Another relevant aspect, highlighted by Chan et al. (2012), consists in the post-unloading phase, when the giver still applies a grasping force even though its sensed load is almost zero. This means that the giver is the agent responsible of the object safety during the passing. The main contribution of the present paper focuses on these two aspects by proposing grip force modulation algorithms, based on the sole load perception including both linear and torsional one, for enabling a safe load sharing and transferring. The building blocks are the slipping avoidance algorithm originally proposed by Costanzo et al. (2020b) and the in-hand pivoting abilities devised by Costanzo et al. (2020a). In the H2R operation the robot gripper is controlled using the slipping avoidance modality so as the grip force is automatically computed based on the sensed load and slipping velocity estimated through the nonlinear observer proposed by Cavallo et al. (2020). However, the slipping avoidance strategy alone revealed ineffective, a communication channel has to be established between the giver and the receiver. We adopt haptic cues applied by the robot to the giver to communicate that the contact with the object is established and its readiness to hold the load. The load transfer takes place safely and effectively without any knowledge of the object inertial properties and center of gravity position with respect to grasping points, but only friction parameters have to be known in advance, mainly related to object surface material. Differently from the approach by Medina et al. (2016), no model learning phase is required.

In the R2H operation the grip release strategy is of paramount importance. Most of the works adopt a simplistic approach, namely, the robot releases the object as soon as a pull by the receiver is detected. However, taking the release decision based on the pulling force only might cause object falls if the receiver is not sharing the load. On the other hand, works deciding the grip release on the load sharing only might be wrong as well since without any pulling force cue there is no clear knowledge of the receiver intention. In this paper, we adopt a strategy based on both indicators, the pulling force and the load share. This greatly enhances the reliability of the R2H handover.

In the R2R operation reliability of the object passing is crucial. Assuming the adoption of parallel grippers only, if the receiver grasps the object in a location too far from the center of gravity, the grip force might exceed the gripper force capability to keep the initial object orientation. In this case the giver should foresee this situation and avoid the grip release. It should communicate to the receiver the need to change the grasp location. We present here a first method to address the problem, based on the anticipatory detection of the slipping velocity. This feature is enabled, in this paper, by a smart use of the slipping detection algorithm by the giver and the controlled sliding by the receiver. Remarkably, in our approach the two robots are independent agents and the controllers are not coordinated but they communicate via haptic cues only.

The rest of the paper is organized as follows. Section 2 describes the reactive control algorithms for visual servoing, slipping avoidance and controlled sliding. Section 3 presents the finite state machines (FSM) handling the whole handover task in the three scenarios R2H, H2R and R2R. Experimental results obtained in an in-store logistics collaborative scenario are described in Section 4, while conclusions are drawn in Section 5.

## 2 Reactive Controllers

In the handover execution, we assume that the receiver, whoever it is, has to adapt to the giver behavior. For instance, in all operation types, the giver shows the object to the receiver in a zone within its field of view, and the receiver has to reach such location even if the giver moves before giving the object. Even in the R2R case this should be achieved because we assume there is no communication between the two control units, apart from the haptic cues during the physical handover. Moreover, any handover task is characterized by a large degree of uncertainty affecting the handover location, object mass and grasp location. We address these issues by resorting to sensor-based reactive control. The first controller is based on visual feedback and is a fast visual servoing algorithm aimed at tracking the object motion carried by the giver. The second controller is based on force/tactile feedback and aims at modulating the grip force so as to avoid object slippage while transferring the load from the giver to the receiver. The two controllers are activated in different times and the scheduling logic is encoded in the state machines described in Section 3.

### 2.1 Visual Servoing Controller

The objective of the visual servoing controller is to generate a velocity of the receiver hand so as to grasp the object to exchange. This feature is certainly needed in the H2R operation, but it can be useful even in the R2R operation in case the base frames of the two robotic agents are not calibrated, as in our experiments.

During the H2R handover, the human giver presents an item to the robot by putting it within the field of view of the camera. Since the human is not able to keep the object fixed or because he/she would like to move the object before passing it, the handover location is time-variant. We address this problem by using a visual servoing controller that tracks the object and controls the robot velocity toward the object location.

The controller is based on the ViSP library (Marchand et al., 2005) and uses the images acquired from an RGB-D camera (RealSense D435i in Figure 1) to adjust the robot pose with respect to the object and correctly grasp it. We acquire offline a target image that corresponds to the desired grasp configuration, then the algorithm moves the robot to align the current image with the target one. The target image implicitly defines the end-effector pose relative to the object, i.e. the grasp pose. The grasp pose of each object is calibrated in a preliminary phase using the gripper with two calibration fingers specifically designed to achieve the desired accuracy of the grasping location. In this paper, for each object there is only one grasp pose, but the same procedure can be repeated to define multiple grasp poses as proposed by Costanzo et al. (2021) for a pick-and-place task. The images are synthetically represented by 3D feature points matched between the target image and the current image by means of a keypoint matching algorithm available in the ViSP library.

The visual servoing algorithm minimizes the error between the target features $s^{\text{*}}$ and the actual ones s:

$$e\left(t\right)=s\left(I\left(t\right),\pi_{i},\pi_{e}\right)-s^{\text{*}}\left(t\right),$$ (1)

where $\pi_{i}$ and $\pi_{e}$ are the intrinsic and extrinsic parameters of the camera, respectively, and I(t) is the actual image. The vectors $s,s^{\text{*}}\in {\mathbb{R}}^{3N_{f}}$, where $N_{f}$ is the number of features, contain the 3D locations of the actual and target features $p_{i}$ and $p_{i}^{\text{*}}$, respectively, thus

$$s={\left[\begin{array}{cccc} p_{1} & p_{2} & \ldots & p_{N_{f}} \end{array}\right]}^{T}$$ (2)

$$s^{\text{*}}={\left[\begin{array}{cccc} p_{1}^{\text{*}} & p_{2}^{\text{*}} & \ldots & p_{N_{f}}^{\text{*}} \end{array}\right]}^{T}.$$ (3)

The algorithm minimizes the error (Eq. 1) by controlling the camera linear and angular velocity with the following law

$$v_{c}\left(t\right)=-\lambda L\left(t\right)e\left(t\right),$$ (4)

where $v_{c}$ is the 6D camera twist, L is the interaction matrix, and λ>0 is the control gain (Marchand et al., 2005).

Commonly, $s^{\text{*}}\left(t\right)={\bar{s}}^{\text{*}}$ is constant. This means applying a step reference to the control algorithm. To ensure stability, the higher initial error imposes to use low gains. This issue is typically overcome by using an adaptive gain which is lower when the error is high and increases as the error declines. Such a solution implies a slower motion in the initial phase. To speed-up the motion, while ensuring stability, we propose to use a high constant gain while applying a time-varying reference $s^{\text{*}}\left(t\right)$ obtained by interpolating each feature between the initial value s(I(0)) and the target one ${\bar{s}}^{\text{*}}$ in a give time $t_{f}$. The interpolation is obtained by applying the same time-varying rigid body transformation to each feature, i.e.,

$${\tilde{p}}_{i}^{\text{*}}\left(t\right)=T\left(t\right){\tilde{p}}_{i}\left(0\right),\;i=1,\ldots ,N_{f}$$ (5)

being $\tilde{p}$ the vector of homogeneous coordinates of the point p. T(t) is a time-varying homogeneous transformation matrix such that T(0)=I and $T\left(t_{f}\right){\tilde{p}}_{i}\left(0\right)={\tilde{\bar{p}}}_{i}^{\text{*}}$. Thus, T(t) is obtained by a linear interpolation between T(0) and $T\left(t_{f}\right)$ in translation and quaternion rotation. This choice implies that the initial error e(0)=0 and e(t) has a bell-like shape.

The homogeneous transformation matrix $T\left(t_{f}\right)$ can be found by solving the following problem

$$\underset{T}{\text{min}}\frac{1}{2}\sum_{i=1}^{N_{f}}{\left\|T{\tilde{p}}_{i}\left(0\right)-{\tilde{\bar{p}}}_{i}^{\text{*}}\right\|}^{2}.$$ (6)

We adopt the Ceres^2^ solver to solve this optimization problem and, to ensure that T is a homogeneous transformation matrix, we parametrize it with a position vector and a unit quaternion.

**FIGURE 2:**
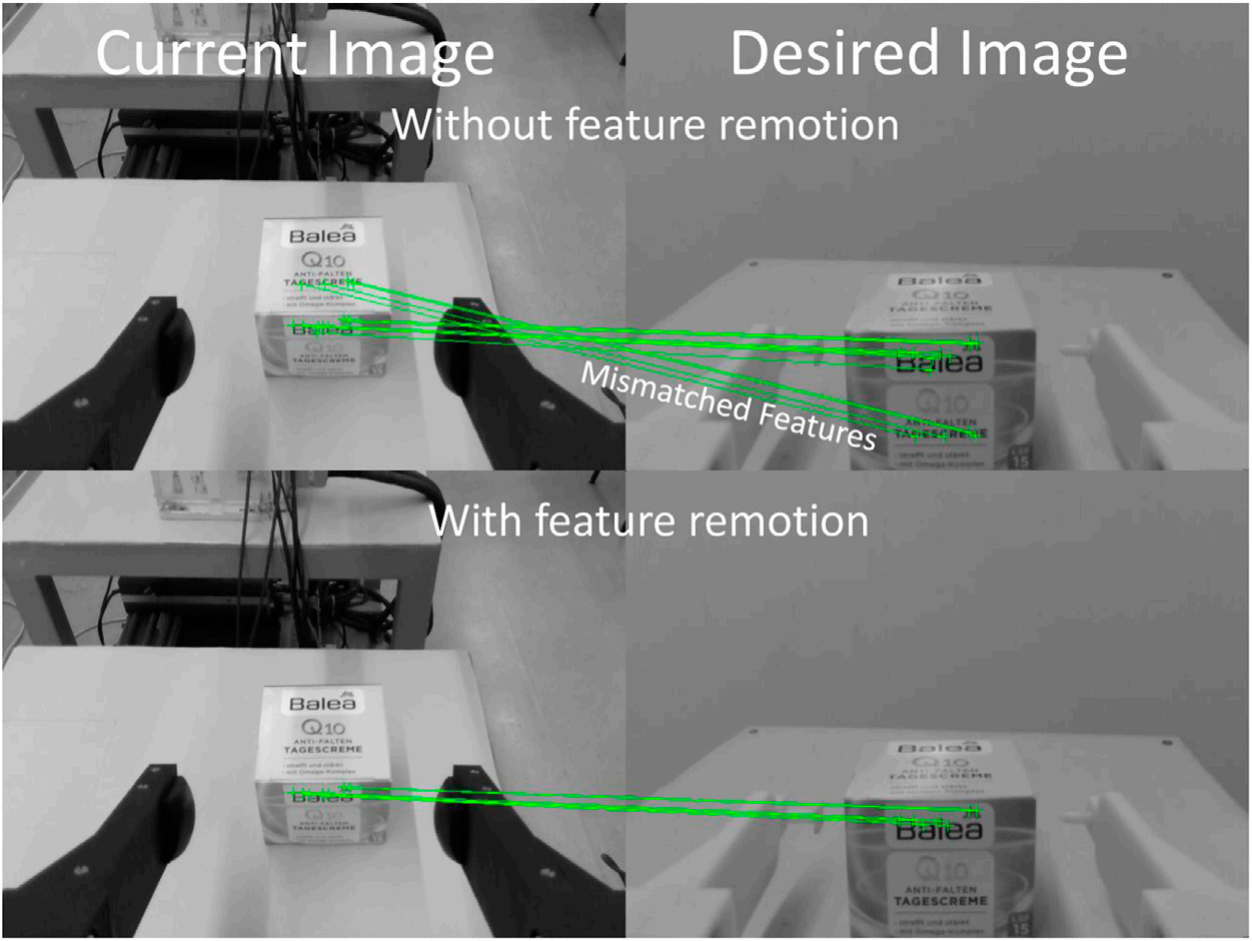
Example of matched features without **(top)** and with **(bottom)** the feature removing algorithm. The images on the right show the 3D printed calibration fingers mounted on the gripper grasping the object in the desired location.

The keypoint matching algorithm of the ViSP library is used to find only the initial features s(I(0)) and the corresponding target features ${\bar{s}}^{\text{*}}$, then the keypoint tracking algorithm of the library tracks the actual feature in real-time yielding s(I(t)). The matching algorithm is based on the texture information of the object and, if the object has the same texture (such as a drawings or text) in two different locations, the matching algorithm could wrongly match two keypoints of these two locations (an example is shown in the top picture of Figure 2). When this happens there is one or more *i* such that the residual in Eq. 6 has a value higher than the required accuracy $\varepsilon_{v}$, i.e.,

$$\exists i:\left\|T{\tilde{p}}_{i}\left(0\right)-{\tilde{\bar{p}}}_{i}^{\text{*}}\right\|>\varepsilon_{v}.$$ (7)

If condition (Eq. 7) is detected, our algorithm does not remove all the features that meet the condition, but it removes only the feature with the highest residual and reiterates the optimization algorithm by solving (Eq. 6) again. This is helpful because in the next iteration, without the removed feature, the optimization algorithm will find a solution with a lower residual on the other features and the condition (Eq. 7) may not be satisfied anymore; in this case the algorithm is stopped. In the end, all the mismatched features are removed (see the bottom picture of Figure 2). The accuracy achieved by the algorithm is relevant to the successful execution of the task by the robot after the handover. For instance, if the robot needs to execute pivoting maneuver, i.e. let the object rotate in-hand so as to reach a vertical orientation, the grasping point needs to be above the CoG otherwise the object would not be vertical at the end of the maneuver. Based on our experience, we estimate that the grasping point should be in a 2 mm ball located on the vertical line above the CoG. For this reason, we set $\varepsilon_{v}=0.002\;\text{m}$ m. We verified that the algorithm is able to achieve such accuracy on the object in Figure 2 which is challenging for the matching algorithm because it has two different faces with very similar textures.

### 2.2 Grip Force Control

**FIGURE 3:**
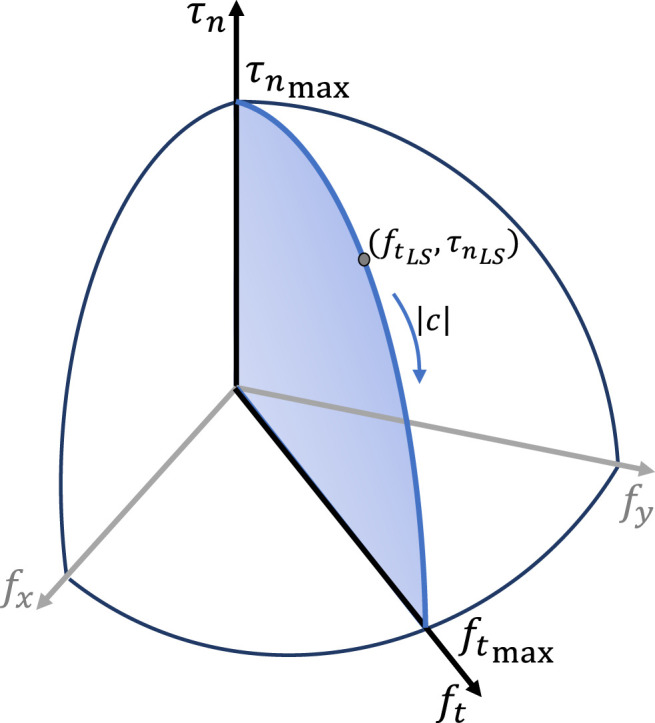
Limit Surface with maximum tangential $\left(f_{t_{\text{max}}}\right)$ and torsional $\left(\tau_{n_{\text{max}}}\right)$ friction; *c* is the position of the instantaneous CoR; $\left(f_{t_{LS}},\tau_{n_{LS}}\right)$ is the maximum external load that the friction can withstand, with the given grip force $f_{n}$.

The control algorithm exploits the data provided by force/tactile sensors mounted on the fingertips, which can measure the 6D contact wrench (Costanzo et al., 2019). Each fingertip is a soft hemisphere with a stiffness and a curvature radius smaller than those of the handled object, hence the contact area is approximately a circle with radius *ρ* and the pressure distribution is axisymmetric. Under these assumptions, the grip force $f_{n}$ is related to the maximum tangential and torsional frictional loads by the relationships

$$\begin{array}{l} f_{t_{\text{max}}}=\mu f_{n},\\ \tau_{n_{\text{max}}}=\mu \xi \delta f_{n}^{\gamma +1}, \end{array}$$ (8)

where *µ* is the dry friction coefficient, depending on the object surface material, ξ is a parameter depending on the pressure distribution, while δ and γ are parameters depending on the soft pad material only. It is well-known that the radius is related to the grip force as $\rho =\delta f_{n}^{\gamma }$ (Xydas and Kao, 1999), where the two parameters can be estimated through an experimental procedure described by Costanzo et al. (2020b). According to the Limit Surface (LS) concept (Howe and Cutkosky, 1996), a sliding cannot occur if the external load $\left(f_{t},\tau_{n}\right)$ applied to the object is internal to the LS (the blue area of Figure 3). The LS can be enlarged by increasing the grip force $f_{n}$. This way, given the measured load $\left(f_{t},\tau_{n}\right)$, it is possible to compute the minimum grip force to hold the object $f_{n_{LS}}$. The method for this computation can be found in (Cavallo et al., 2020). However this grip force can balance constant loads only, while in dynamic conditions, i.e., with time-varying loads and in case of non negligible accelerations, an additional grip force is needed. This can be computed, based on a dynamic model of the instantaneous rotational motion of the object about the Center of Rotation (CoR), via the nonlinear observer

$$\dot{\zeta }=\omega -\frac{\sigma_{0}}{g\left(f_{n},c\right)}\zeta \left|\omega \right|$$ (9)

$$\dot{\omega }=l\left(-\sigma_{0}\zeta -\sigma_{1}\left(f_{n},c\right)\omega +y\right),$$ (10)

where ζ is the internal LuGre state (Canudas de Wit et al., 1995) and ω is the estimated slipping velocity. The function $g\left(f_{n},c\right)=\left|cf_{t_{LS}}\right|+\left|\tau_{n_{LS}}\right|$ represents the maximum dry friction torque about the CoR, it depends on the normal load and on the CoR position *c*, which can be estimated via an algorithm that can be found in (Cavallo et al., 2020). The term $\sigma_{0}\zeta$ is the actual dry friction being $\sigma_{0}$ the stiffness of the microasperities of the contact surfaces; $y=\tau_{n}-cf_{t}$ is the generalized torque measured at the fingertip about the CoR axis and *l* is the observer gain. As soon as $\left|y\right|>g\left(f_{n},c\right)$, a slipping velocity ω builds up. The viscous friction function $\sigma_{1}$ depends on the area of the contact surface, i.e., (Costanzo, 2020)

$$\sigma_{1}\left(f_{n},c\right)=\pi \rho^{4}\beta_{a}\left(\frac{c^{2}}{\rho^{2}}+\frac{1}{2}\right),$$ (11)

where $\beta_{a}$ is the viscous friction coefficient per area unit (Shkulipa et al., 2005), that can be estimated with the same exploration procedure used to estimate the dry friction coefficient μ, i.e., by rubbing the object at constant velocity.

The methods described above are used to design a grasp controller for computing the grip force to avoid object slippage, the so-called slipping avoidance (SA) control modality, used in the handover strategy described in Section 3. The grip force is the superposition of two components, namely,

$$f_{n}=f_{n_{LS}}+f_{n_{d}},$$ (12)

where $f_{n_{LS}}$ and $f_{n_{d}}$ are the so-called static and dynamic components, respectively. $f_{n_{d}}$ is aimed at counteracting time-varying external disturbances, such as the inertial forces that arise during the giver release and object transportation phases. It is computed by a linear control action on the estimated slipping velocity *ω* with the aim to regulate it to zero. The two actions can be activated separately depending on the specific phase of the handover task. When both actions are present the control mode is called dynamic mode, when the dynamic action is switched off, the control mode is called static mode. The activation strategy of the two modes is described in detail later on in the paper. Moreover, to ensure that a contact is always present so as the tactile sensors are able to measure, when the SA is active, the grip force is lower bounded by a minimum value depending on the accuracy and signal to noise ratio of the force/tactile sensor.

In the pre-handover phase, another control modality can be useful to change the relative orientation between the gripper and the object in a configuration that is more suitable for the exchange. This modality is called *pivoting* and its aim is to bring and keep the object in a vertical position, i.e., with zero frictional torque. Again, the LS method can show that the grasp force to achieve this objective coincides with the Coulomb friction law, i.e., $f_{n}=f_{t}/\mu$. Thus, the grasp force is brought from its current value to this limit by applying the first order linear filter in the discrete time *k*

$$f_{n}\left(k+1\right)=\left(1-\alpha \right)f_{n}\left(k\right)+\frac{\alpha f_{t}\left(k\right)}{\mu },$$ (13)

where the value α sets the time constant of the filter and $f_{t}\left(k\right)$ is the measured tangential force. The value of α changes the velocity of the pivoting maneuver and should be chosen depending on the bandwidth of the available force-controlled gripper. The interested reader can find more details about the algorithm in (Costanzo et al., 2020b; Cavallo et al., 2020).

## 3 Handover Strategy

The assumptions underpinning the proposed approach and the requirements of the application tackled in this paper are summarized as follows:

• We do not investigate the signaling problem, we assume that the receiver already agreed on getting the object from the giver
• The receiver already knows which object the giver is passing;
• Both the agents have the capability to withstand the weight of the object;
• The objects to exchange are texture-rich and with a cylinder-like or parallelepiped-like shape;
• A simple handover protocol is known to the agents based on haptic cues;
• We use parallel grippers;
• The handover location is decided by the giver;
• We are not allowed to sensorize, e.g., using ARTags, neither the objects or the human;
• We are allowed to use only sensors on board the robot, no external sensor can be used;
• In the R2R case no communication between the robot control units is allowed.

Under these assumptions, which seem reasonable in the in-store logistics settings where performance and cost should be well balanced, with only visual and tactile information we perform natural handover operations.

**FIGURE 4:**
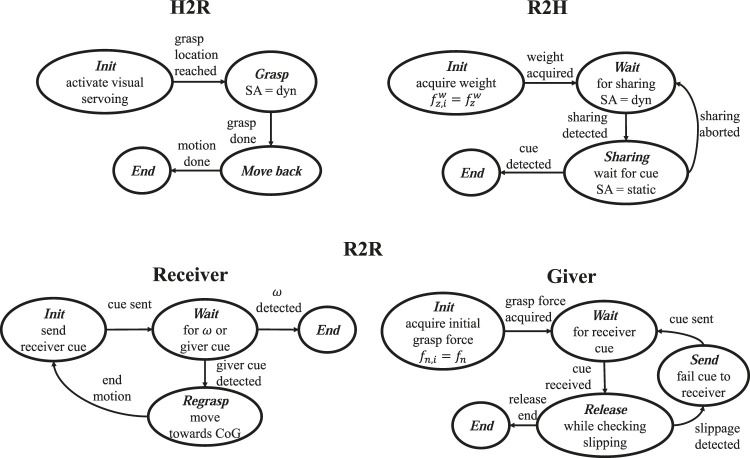
FSMs for the handover algorithms: name of the states in boldface italic.

For each handover operation, H2R, R2H and R2R, we use different strategies. Each one corresponds to a FSM represented in Figure 4 and described hereafter.

### 3.1 H2R Operation

In the H2R operation, the human holds an item and the robot has to grasp it from the human hand. The strategy is represented in the top-left FSM diagram of Figure 4. In the *Init* state the robot activates the visual servoing algorithm, described in Section 2.1, to reach the handled object in the grasp configuration corresponding to the target image. During this phase, the human can move and the robotic receiver tracks the object by complying with the dynamic behavior of the giver. When the time varying feature trajectory (Eq. 5) terminates and the visual servoing error norm is below the desired accuracy $\varepsilon_{v}$, the grasp location is considered reached and the gripper is commanded to close the fingers. As soon as contact is established with the minimal grip force, the visual servoing controller is disabled and the SA is activated in dynamic mode (Grasp state). Note that, during the gripper closing (before the SA activation), the visual algorithm is still active because the human operator can move the object, which makes the handover operation fluid. As soon as the SA is activated, the robot is commanded to move backward (Move back state), this gives a haptic cue to the human that feels a tangential force in the direction of the robot displacement, this cue means that the robot has securely handled the object and the human can release it.

### 3.2 R2H Operation

In the R2H operation, a robot intends to pass an held object to a human. The algorithm is described by the top-right FSM diagram of Figure 4. During the *Init* state the system is in the pre-handover phase, the gripper is controlled in SA dynamic mode, the robot feels the object weight by means of the sensorized fingertips; the weight is represented by the force component $f_{z}^{w}$ that the sensor measures along the *z*-axis of the world frame (assumed aligned and opposed to the gravity). Note that, before activating the SA mode, the gripper is first controlled with a pivoting mode in order to present the object to the receiver with a vertical orientation. This expedient allows the receiver to grasp the object with no torsional load, thus requiring a minimal effort in terms of grip force. Once the weight $f_{z,i}^{w}$ is acquired, the robot waits for the human to initiate the physical handover phase (Wait state). During the handover, the object is held by both the robot and the human and the load is shared by the two agents. In the Wait state, the slipping avoidance algorithm is in dynamic mode (Eq. 12) and it counteracts any disturbance, i.e., any external force or torque applied to the object. When the human receiver starts grasping the object holding part of the load, the robot feels less weight and it enters into the Sharing state. This is done by comparing the initial measured weight $f_{z,i}^{w}$ with the actual force component $f_{z}^{w}$, by checking the condition

$$f_{z}^{w}>\nu_{s}f_{z,i}^{w},\;0<\nu_{s}<1,$$ (14)

where the scale factor $\nu_{s}$ establishes the amount of weight the human has to withstand before the robot enters the sharing state. In other words, when condition (Eq. 14) is satisfied, the robot assumes that the human has the intention to share the object weight and thus it believes that the handover can take place safely. Note that the sign of the inequality takes into account the direction of the *z*-axis, opposed to the gravity, thus the measured force $f_{z,i}^{w}$ is negative. In the Sharing state the system is in the physical handover phase and the human is helping the robot to hold the object. According to the feedback given by different subjects during several trials, we decided to switch off the SA dynamic control action (static mode) because the subjects complained for an annoying vibration felt during the load sharing caused by the controller reaction to the slight trembling of the receiver. In this state, the FSM takes into account the possibility that the human does not have the intention to take the object anymore. This event is detected, similarly to (Eq. 14), by checking the condition

$$f_{z}^{w}<\nu_{w}f_{z,i}^{w},\;0<\nu_{w}<1,$$ (15)

where the scale factor $\nu_{w}>\nu_{s}$ establishes the amount of weight the robot has to withstand again before returning in the wait for the Sharing state. In addition, to avoid useless switches between the Waiting and the Sharing states, the parameters $\nu_{s}$ and $\nu_{w}$ are selected such that $\left(\nu_{w}-\nu_{s}\right)\left|f_{z,i}^{w}\right|$ is greater than the noise level affecting the measured force, being $\left|f_{z,i}^{w}\right|$ the weight of the lightest object. When the condition (Eq. 15) is verified, the FSM returns into the Wait state. Still in the Sharing state, the FSM waits for a haptic cue informing the robot that the human is pulling the object. Once again, this is done by measuring the forces at the fingertips. When the cue is detected, the robot opens the gripper and the handover is complete. The haptic cue is detected if the following condition holds

$$f_{z}^{w}>\psi_{z}\vee f_{\text{pull}}>\psi_{p},\;\psi_{z},\;\psi_{p}>0,$$ (16)

where $f_{\text{pull}}$ is the measured external force along the pulling direction, defined as the projection of the *y* axis of the grasp frame on the xy-plane of the world frame (see the frame in the left picture of Figure 1). The first part of the condition means that the receiver is pulling the object upwards, while the second part means that the receiver is pulling the object out of the gripper (note that in case of a pushing force, $f_{\text{pull}}$ is negative). The parameters $\psi_{z}$ and $\psi_{p}$ are thresholds selected by testing the algorithm using various threshold values with 10 human volunteers (5 male and five female) that gave us feedback on their sensation during the pulling phase. It is important to underline that the condition (Eq. 16) is checked only in the Sharing state, this implies that the giver releases the object only if the receiver shares a sufficient amount of weight and he/she pulls the object. If a pulling force is applied without any load sharing, the robot keeps the object firmly with the SA grasp control.

### 3.3 R2R Operation

The handover strategy for the R2R case is executed by two FSMs, one for the giver and one for the receiver, depicted in the bottom diagram of Figure 4. One might think to reuse the same strategy devised for the H2R case for the robot receiver and the same strategy devised for the R2H for the robot giver. However, this approach cannot handle the case the selected grasp configuration to be achieved via the visual servoing is not compatible with the maximum grip force of the receiving gripper. This might happen if the grasp pose is such that the gravity torsional load requires a grip force higher than the gripper capacity, i.e. the grasp location is too far from the center of gravity. When the giver is a human, this exception is easily handled by the giver exploiting his/her manipulation dexterity and the ability to coordinate visual and tactile feedback to anticipate the failure event. Therefore, in the R2R case we use the tactile feedback of the giver, in charge of the object safety, to anticipate the slipping event that would happen if the receiver grip force, required by the slipping avoidance, exceeded the gripper limit. Exploiting the a priori knowledge contained in the soft contact model and the tactile perception data only, the giver is able to foresee this failure event and reacts to avoid it, still using haptic communication only, as detailed in the following.

The giver in the *Init* state is grasping an object with the grasp control in SA mode, then it records the grip force $f_{n,i}$ necessary to hold the object in the current configuration. In this pre-handover phase, the giver brings the object inside the field of view of the receiver camera, so that this starts tracking the object to grasp it. The giver is now waiting for a haptic cue provided by the receiver that has the intention to take the object (Wait state). The receiver executes a short backward and forward motion by first pulling the object to generate a pulling force $f_{\text{pull},g}$ detected by the giver fingers, and then it returns to the initial position, so as to avoid keeping an external load that the giver grip force counteracts. The detecting condition of the cue is

$$f_{\text{pull},g}>\psi_{p},$$ (17)

where the threshold $\psi_{p}$ is the same used in the R2H case. The giver is now in the *release* state and it starts opening the gripper by setting zero grasp force with a rate of 0.3 N/s, value tuned in combination with the response time of the slipping velocity observer. While the grip force is decreasing, the robot checks if the grip force reached zero and the following condition

$$\left|\vartheta_{g}\right|=\left|\int_{0}^{t}\omega_{g}\left(\eta \right)\text{d}\eta \right|>\psi_{\vartheta },$$ (18)

where $\omega_{g}\left(t\right)$ is the object velocity with respect to the giver fingertips estimated by the observer in (Eq. 9), (Eq. 10) with the measured torque *y* on the giver gripper. $\psi_{\vartheta }$ is a threshold that establish the maximum rotational slippage that can be accepted for the execution of task following the handover operation. Such a condition is aimed at detecting a slipping event of the commonly held object. Hence, to avoid actual slippage it is sufficient to use in the observer a value of the friction coefficient *µ* slightly lower than the actual one. This corresponds to consider a lower maximum friction torsional moment $g\left(f_{n},c\right)$ and, in turn, a contracted LS, i.e., a virtual LS instead of the real one. This causes an estimated virtual slipping velocity that anticipates the slipping event, which would happen by continuing to reduce the grip force. This means that the actual load would cause a slippage at the giver contact surfaces which indirectly indicates that the receiver cannot safely withstand the load. This way $\vartheta_{g}$ takes on the meaning of a virtual sliding angle. If the first condition on the grip force is verified before condition (Eq. 18), then the task of the giver ends, since the object is fully held by the receiver because no significant slippage occurred during the gripper opening. On the other hand, if condition (Eq. 18) holds with a non zero grip force, it means that a significant (virtual) slippage is happening and thus the receiver is trying to hold the object in a wrong configuration. Then, the giver grasp control is set to SA mode again, the robot giver sends a haptic cue (short forward and backward motion) to inform the receiver of the likely failure and, finally, it goes back to the Wait state with a grasp control that keeps the grip force to the initial one $f_{n,i}$. From this state, different recovery strategies can be devised, e.g., re-plan the receiver grasp. In this paper, where we focus on the possibility to catch an exception only using tactile feedback and a model, we assume to know a new grasp configuration for the receiver compatible with the gripper capability and the receiver will move toward this new grasp location.

The robot receiver in the *Init* state, with the grasp control in SA mode, sends a haptic cue to the giver, again with the short backward and forward motion. Then, it comes to a Wait state where the following conditions are checked:

$$f_{\text{pull},r}<-\psi_{p}$$ (19)

$$f_{\text{pull},r}\geq -\psi_{p}\wedge \left|\omega_{r}\right|>\psi_{\omega },$$ (20)

where $f_{\text{pull},r}$ and $\omega_{r}$ are the pulling force and the slipping velocity detected by the receiver, respectively, $\psi_{\omega }$ is the threshold on the slipping velocity necessary to detect the slipping event. If the first condition is verified, the receiver detects the cue sent by the giver informing it of the possible failure, thus it enters the Regrasp state to handle this exception. In our case we move the receiver toward the giver to grasp the object closer to the center of gravity. This way, a lower grip force is necessary to hold the object in its orientation, hence the receiver can try again to take the object from the giver re-starting from the *Init* state. If the second condition, alternative to the first one, holds, then the handover is successful and the task ends.

## 4 Experiments

The handover algorithms described so far have been tested in a number of experiments carried out on the setup described in Section 4.1, testing all three handover operations H2R, R2H and R2R.

### 4.1 Experimental Setup

The experimental setup is composed of two robots (Figure 1), a Kuka LBR iiwa seven equipped with a WSG-50 gripper, and a Yaskawa Motoman SIA5F equipped with a WSG-32 gripper. The finger motion of both grippers is velocity controlled via a ROS interface that communicates with the grippers through a LUA script running on the gripper MCU. Because of the LUA interpreter, the velocity commands can be sent to the grippers only with a rate of 50 Hz, this limits the performance of the grasp force controller. Each gripper is equipped with two SUNTouch sensorized fingertips (Costanzo et al., 2019) that are able to measure the 6D contact wrench. The method to extract the external wrench applied to the object from the single finger contact wrench vectors is explained in (Costanzo et al., 2020b). The sensors communicate with the ROS network via a serial interface at a sampling rate of 500 Hz. The mean error on the measured force is 0.2 N, hence we set the lower bound of the SA algorithm to a conservative value of 0.5 N. The grip force is controlled via a low-level force loop that acts on the finger velocity to track a reference grip force with a typical response time of 0.15 s, hence the time constant of the filter in (Eq. 13) is set to 0.33 s corresponding to the value of α reported in Table 1 considering the sampling rate of 500 Hz of the digital implementation. The iiwa robot is also equipped with an Intel D435i RGB-D camera, mounted in an eye-in-hand configuration, used for the visual servoing phase.

**TABLE 1:** Parameters of the algorithm (SI units). The friction model parameter *μ*, *δ*, and *γ* appear in Eq. 8 and $\beta_{a}$ in Eq. 11. Note that: object mass is not used in the algorithms, it is reported for the reader’s convenience in the interpretation of the results; the parameter δ is object independent for rigid objects, for partially deformable objects it has to be estimated for each object; object B is the same as object A but the bottle is empty; the parameter α is reported for a sampling rate of 500 Hz.

Object dependent parameters											
		Object A 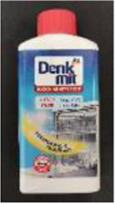		Object B 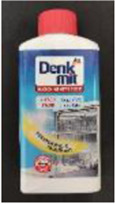			Object C 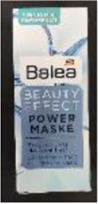		Object D 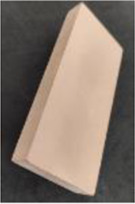		Object E 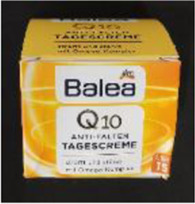
							iiwa	SIA5F	iiwa	SIA5F	
*μ*		0.85		0.85			0.57	0.65	0.60	0.65	0.60
*β*_a_		3.47 10^8^		3.47 10^8^			2.61 10^8^	4.61 10^8^	3.47 10^8^	1.73 ⋅10^9^	3.47 10^8^
δ		1.83 10^–3^		1.83 10^–3^			1.83 10^–3^	4.80 10^–3^	1.83 10^–3^	3.8 10^–3^	1.83 10^–3^
Mass		0.290		0.103			0.082		0.099		0.172

Object independent parameters											
*l*	*σ*_0_	*α*	γ	λ	ϵ_*v*_	*ν*_*s*_	*ν*_*w*_	*ψ*_*z*_	*ψ*_*p*_	*ψ*_*ϑ*_	*ψ*_*ω*_
4,000	50	0.006	0.254	1.0	0.002	0.5	0.98	1.0	0.6	0.087	0.05

Two sets of experiments have been carried out. The first concerns H2R and R2H handover operations performed in a typical pick-and-place task in a collaborative shelf replenishment scenario of a retail store. The setting consists of shelf layers with a clearance of 15 cm. Each shelf has different facings to place the objects detached by 5 cm height separators. A human operator hands an object over to the iiwa robot (H2R), which then places it on a shelf layer. The placing motion is autonomously planned off-line using the method proposed by Costanzo et al. (2021) and the solution found to accomplish the task might include pivoting operations (pivoting the object inside the fingers or rotating the gripper about the grasping axis while keeping the object fixed). The method allows the robot to plan off-line even if the starting robot configuration is likely different at run time since the object is passed by a human and it is not picked from a fixed location. After the place task, the robot is asked to re-pick the same object and hands it over to the human (R2H). This sequence has been selected simply because it allows us to test both handover operations with a human.

The second set of experiments concerns the R2R handover operation. The SIA5F robot is commanded to pick an object in a given position and to pass it to the iiwa robot. The SIA5F is controlled according to the R2R Giver FSM, while the iiwa is controlled via the R2R Receiver one. The two robots communicate only via haptic feedback through the protocol defined in the FSMs. The R2R experiments have been carried out with two different objects.

The quantitative results reported in the next sections refer to experiments carried out with three different objects, a plastic bottle full of liquid (Object A), the same bottle but empty (Object B), and a cardboard box (Object C). Moreover, in the Supplementary Video S1, there are an extra R2R experiment performed with a resin block (Object D) and an extra H2R experiment carried out with a cardboard cube (Object E). Object D has been used since it is fully rigid and is made of the same material used in the calibration of the tactile sensors, hence the experiment runs in ideal conditions highlighting details of the physical handover phase. Object E is challenging to handover, since it is small compared to the robot fingers and the human should present it in a way depending on the pick-and-place task, in particular on the placing action the robot has to do. Table 1 shows all the objects used in the experiments with their friction parameters together with all parameters necessary to run the algorithms that do not depend on the specific object. Note that different friction parameters have been used for iiwa and SIA5F robots since the fingers are different.

### 4.2 H2R and R2H Experimental Results

**FIGURE 5:**
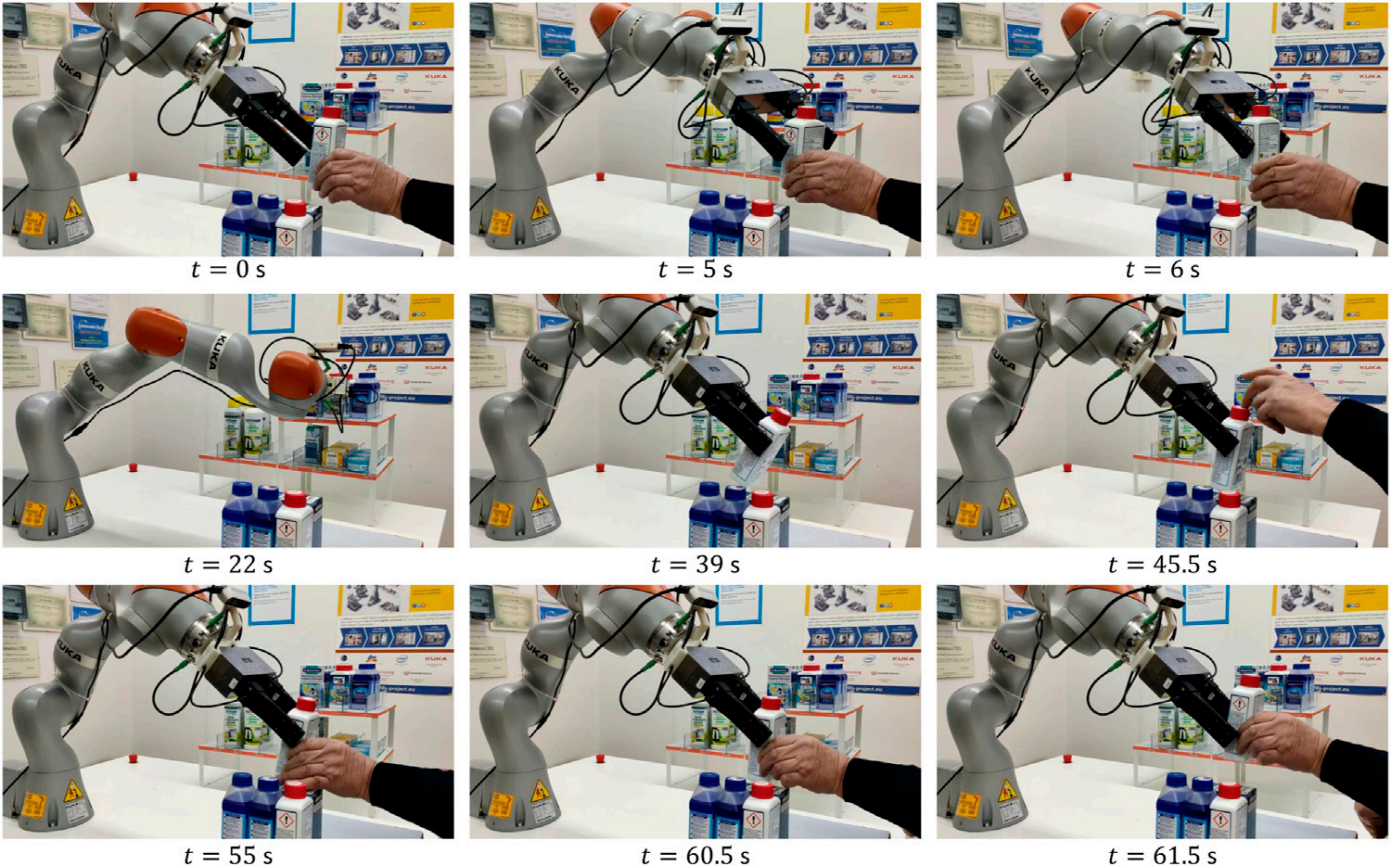
First experiment (object A): H2R handover operation **(top row)**, object placing phase **(middle row)**, R2H handover operation **(bottom row)**.

**FIGURE 6:**
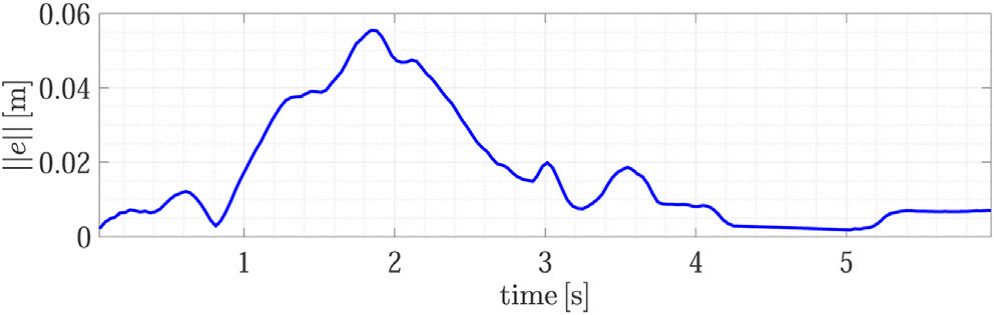
First experiment (Object A): visual servoing tracking error during the H2R operation.

Figure 5 shows the snapshots of the first experiment execution. In the beginning, the human brings the object A inside the field of view of the robot camera and the robot goes toward it by using the visual servoing algorithm (*t* = 0 s). Before the grasp, the human rotates the object (*t* = 2 s) by changing its pose during the robot motion to reach the object. We intentionally introduced this dynamic change to show the capability of the algorithm to reach the target grasp point (*t* = 6 s) even in case of rapid motions of the human giving the object to the robot. This feature alleviates the cognitive burden of the giver, who does not need to focus on staying firm in the handover location waiting for the robot. Figure 6 shows the visual servoing error during this phase, the error starts from zero with a bell-like shape thanks to the time-varying reference features $s^{\text{*}}\left(t\right)$ described in Section 2.1. This strategy permits a fast (but still smooth) convergence of the algorithm. Between *t* = 3 s and *t* = 4 s the error variation corresponds to the object rotation by the human, after that the error decreases and then, around *t* = 5 s the human moves again just before the robot grasp and the error increases, but still the grasp is successful. The error does not decrease again because the contact has been established and the physical handover phase begins.

**FIGURE 7:**
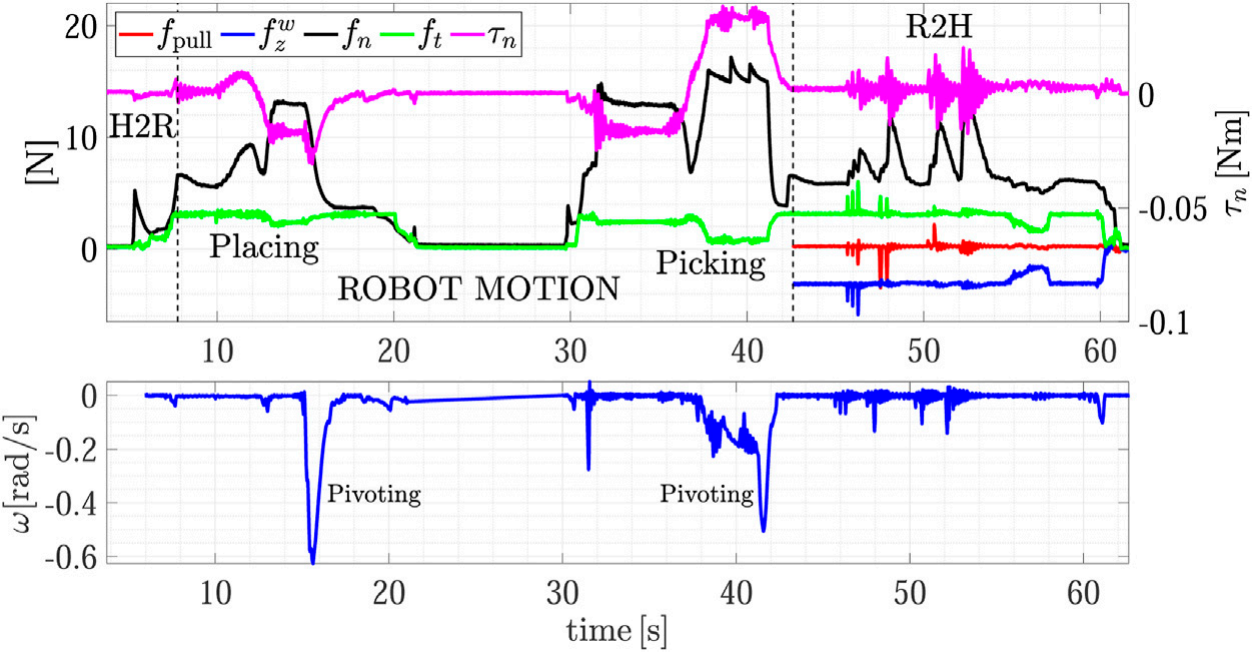
First experiment (Object A): forces and torsional moment measured by the robot during the whole task execution **(top plot);** estimated slipping velocity **(bottom)**.

**FIGURE 8:**
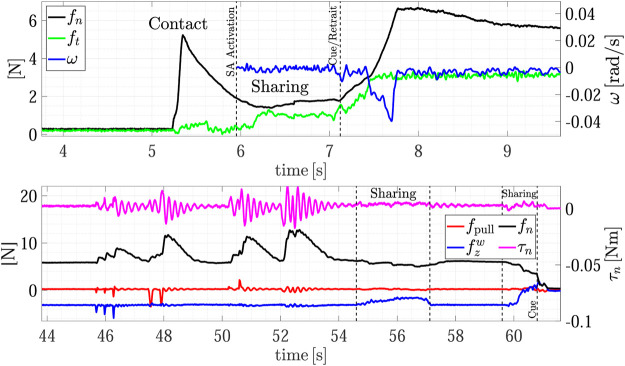
First experiment (Object A): detail of the H2R physical handover phase **(top)**; detail of the R2H physical handover phase **(bottom)**.

Figure 7 shows the forces and torsional moment as well as the estimated sliding velocity during the whole experiment, while the top plot of Figure 8 shows a detail of the H2R physical handover phase. The first grasp force peak depends on the impact velocity between the fingers and the object, then at *t* = 6 s the SA mode is activated and the receiver automatically chooses the grasp force. Here the load sharing phase starts, and both the agents hold the object weight. The weight of object A is 290 g, but in the sharing phase (around *t* = 6.5 s) the robot feels a tangential load $f_{t}$ corresponding to less than half of the weight (107 g), and, in turn, the slipping avoidance algorithm applies a grasp force of about 1.5 N, demonstrating how sensitive is the grip force modulation implemented by the SA controller. At about *t* = 7 s the robot moves back to give a haptic cue to the human giver. The human releases its grasp and the robot withstands all the weight as shown in Figure 8-top. The duration between the end of the sharing phase (when the robot gives the cue) and the end of the object passing (when all the load is withstood by the robot) is about 400 ms, that is in line with the passing time observed in Human-to-Human handovers (Mason and MacKenzie, 2005; Chan et al., 2012; Endo et al., 2012). During the passing phase we observe two peaks in the velocity profile, a first smaller peak at about *t* = 7.1 s corresponds to the robot cue, then, at about *t* = 7.5 s there is another peak that corresponds to the giver release. The velocity increases because the robot accelerates to go in the placing pose. The pattern of the slipping velocity which does not go to zero between the giver release and the robot acceleration phases indirectly demonstrates the fluidity of the robot motion without dead times. After the handover, all the weight is withstood by the robot, the sensors measure a tangential force $f_{t}$ of about 3.1 N including both the weight and the inertial forces, and the slipping avoidance algorithm regulates the grasp force at about 5 N.

After the H2R phase the robot moves to place the object on the top shelf from *t* = 7.5 s to *t* = 22 s (see also Figure 5). The robot motion causes a variation of the gravity torsional load on the fingertips, this, in turn, causes an increment of the grasp force applied by the slipping avoidance algorithm. As shown in the Supplementary Video S1, at the end of the placing action, the robot executes a pivoting maneuver, autonomously planned, to reorient the object in a vertical configuration to correctly place it. This event is visible in the estimated slipping velocity signal (see the bottom plot of Figure 7) at about *t* = 16 s where the observer catches the velocity profile corresponding to the pivoting maneuver.

The same experiment presents also the dual case, where the robot takes an object from the shelf and gives it to a human. At about *t* = 30 s the robot picks again the object from the shelf and presents it to the human operator according to the strategy encoded in the R2H FSM. As shown in the Supplementary Video S1, before activating the FSM, the robot performs a pivoting maneuver to present the object in a vertical configuration, this way all the gravity torque accumulated during the robot motion vanishes (see Figure 7, *t* = 42 s).

The results of the R2H phase are detailed in the bottom plot of Figure 8. From *t* = 44 to *t* = 54 s the FSM is waiting for the Sharing state and counteracts the external disturbances intentionally applied by the human who touches repeatedly the object held by the robot. The plot shows the two signals used in the FSM, i.e., the pulling force $f_{\text{pull}}$ and the force aligned with the gravity vector $f_{z}^{w}$. During this time interval, the operator applies disturbances along $f_{\text{pull}}$, $f_{z}^{w}$ and on the torque $\tau_{n}$, without entering in the *Sharing* state. The SA algorithm counteracts these disturbances by modulating the grip force $f_{n}$. At *t* = 54.5 s the human holds the object and partially withstands its weight. The robot feels this event because $f_{z}^{w}$ decreases and the FSM enters in the Sharing state since condition (Eq. 14) is verified. At *t* = 57 s, the human decides to not get the object and releases it without pulling, thus the measured weight $f_{z}^{w}$ increases again and, automatically, due to the SA mode, the robot increases the grasp force again. Finally, at about *t* = 59.6 s the human holds the object again and the FSM enters in the Sharing state again. This time, the human chooses to take the object and applies a pulling force in the direction of $f_{z}^{w}$ giving a cue to the robot, detected by checking condition (Eq. 16), which releases the grasp in about 360 ms.

**FIGURE 9:**
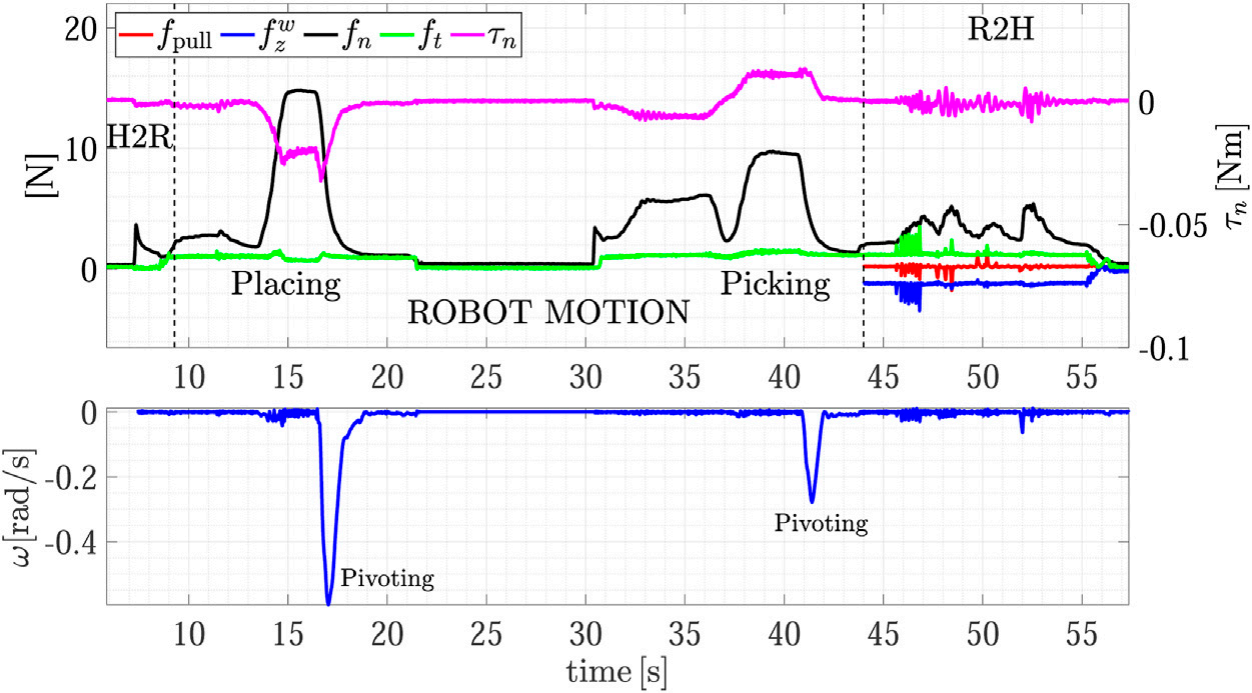
Second experiment (Object B): repetition of the first experiment using an empty bottle; forces and torsional moment measured by the robot during the whole task execution **(top plot);** estimated slipping velocity **(bottom)**.

**FIGURE 10:**
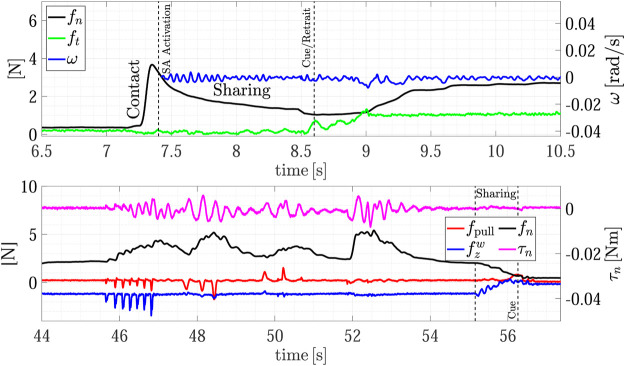
Second experiment (Object B): detail of the H2R physical handover phase **(top)**; detail of the R2H physical handover phase **(bottom)**.

The same experiment has been repeated with the same bottle but now empty (Object B). It is worth mentioning that no vision system is able to estimate the weight of a non-transparent closed bottle and without the force/tactile sensing is not possible to distinguish between Object A and Object B. The experiment description is identical to the previous one and the full task is reported in Figure 9. The top plot of Figure 10 shows the detail of the H2R phase. The plot is qualitatively similar to the previous experiment, but now the measured tangential force and the resulting grasping force are lower. In particular, at the end of the H2R the robot feels a tangential force of about 1 N which corresponds to the object weight of 103 g. In turn, the grasping force $f_{n}$ is automatically set by the slipping avoidance algorithm to a value of 2.4 N which depends on the friction coefficient *µ*. The bottom plot of Figure 10 shows the detail of the R2H phase. Once again, from *t* = 44 to *t* = 54 s, the R2H FSM is in the *Wait* state and counteracts the disturbances on the measured force and torque. At *t* = 55 s the human holds the object and the FSM enters in the *Sharing* state. Then, the human decides to keep the object giving the haptic cue to the robot by pulling it. As soon as the condition (Eq. 16) is verified, at *t* = 56.2 s, the robot releases the object.

**FIGURE 11:**
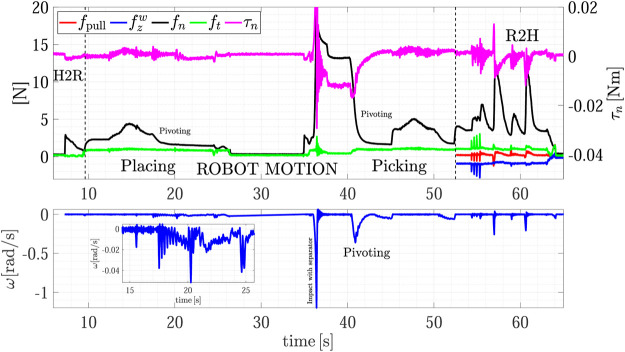
Third experiment (Object C): forces and torque measured by the robot during the whole task execution **(top plot);** estimated slipping velocity **(bottom)**.

**FIGURE 12:**
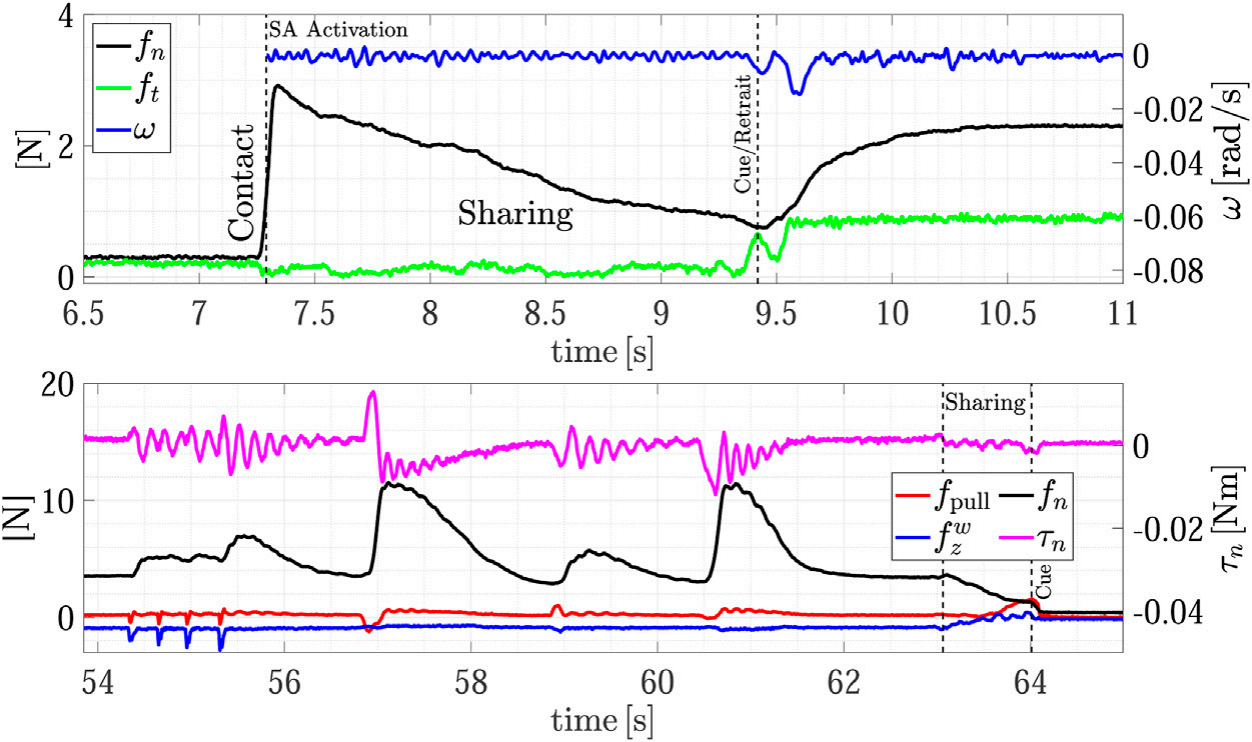
Third experiment (Object C): detail of the H2R physical handover phase **(top)**; detail of the R2H physical handover phase **(bottom)**.

The third experiment of this subsection involves object C and it is reported in Figure 11. The H2R and R2H handover phases (Figure 12) are very similar to those of the previous experiments and we will not discuss them further. This experiment is presented to show the particular pick-and-place task, because the handover grasp configuration is not compatible with the place location due to the tight clearance between two shelves. As shown in the Supplementary Video S1, to execute the placing maneuver the robot uses the gripper pivoting ability, again autonomously planned, i.e., the object remains fixed while the gripper rotates about the grasping axis. This happens from *t* = 20 to *t* = 25 s (see the estimated sliding velocity in the zoom of the bottom plot of Figure 11). After the placing the robot retreats. Then, it begins the new pick-and-place task to make the R2H operation. The robot picks the object again and, during its motion, the object impacts the facing separator. This is caught by the slipping avoidance algorithm that estimates a velocity peak at *t* = 36.4 s and, in turn, increases the grasping force to counteract the generated torsional moment $\tau_{n}$, as evident in the top plot of Figure 11. The rest of the task phases are the same of the previous experiments.

### 4.3 R2R Experimental Result

**FIGURE 13:**
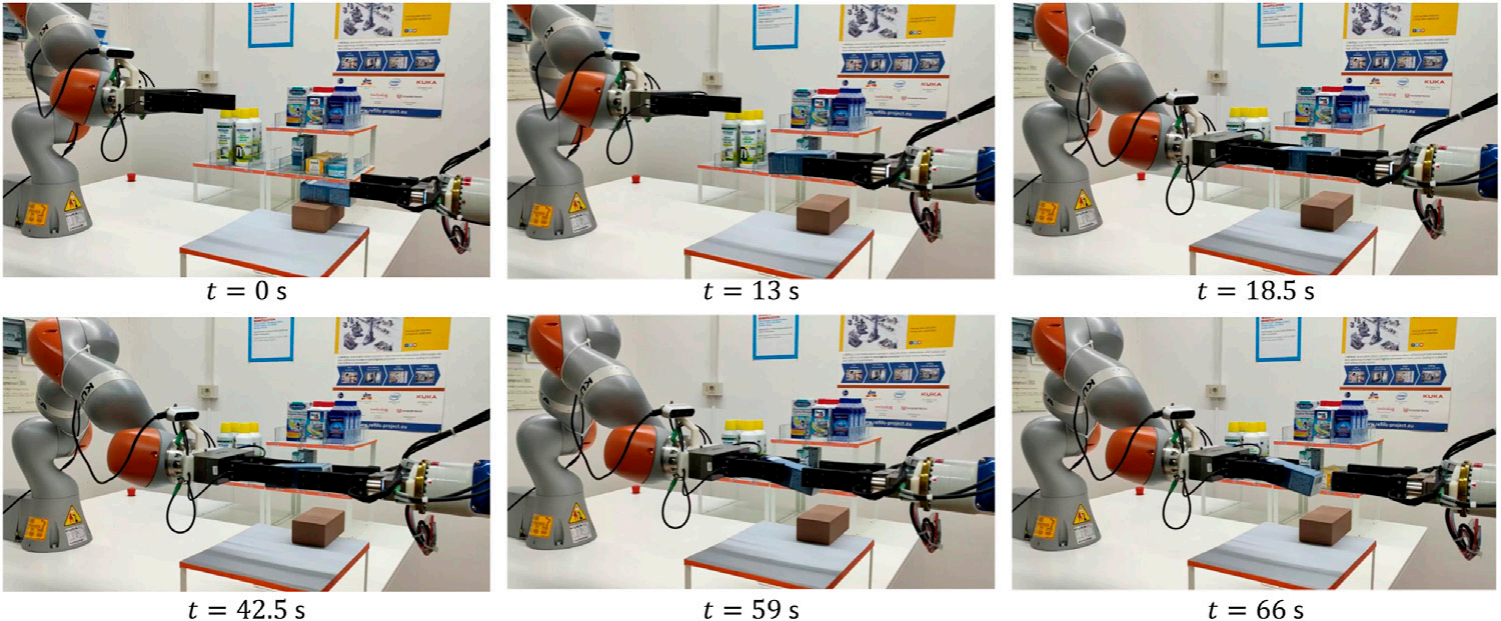
Fourth experiment (object C): R2R operation.

**FIGURE 14:**
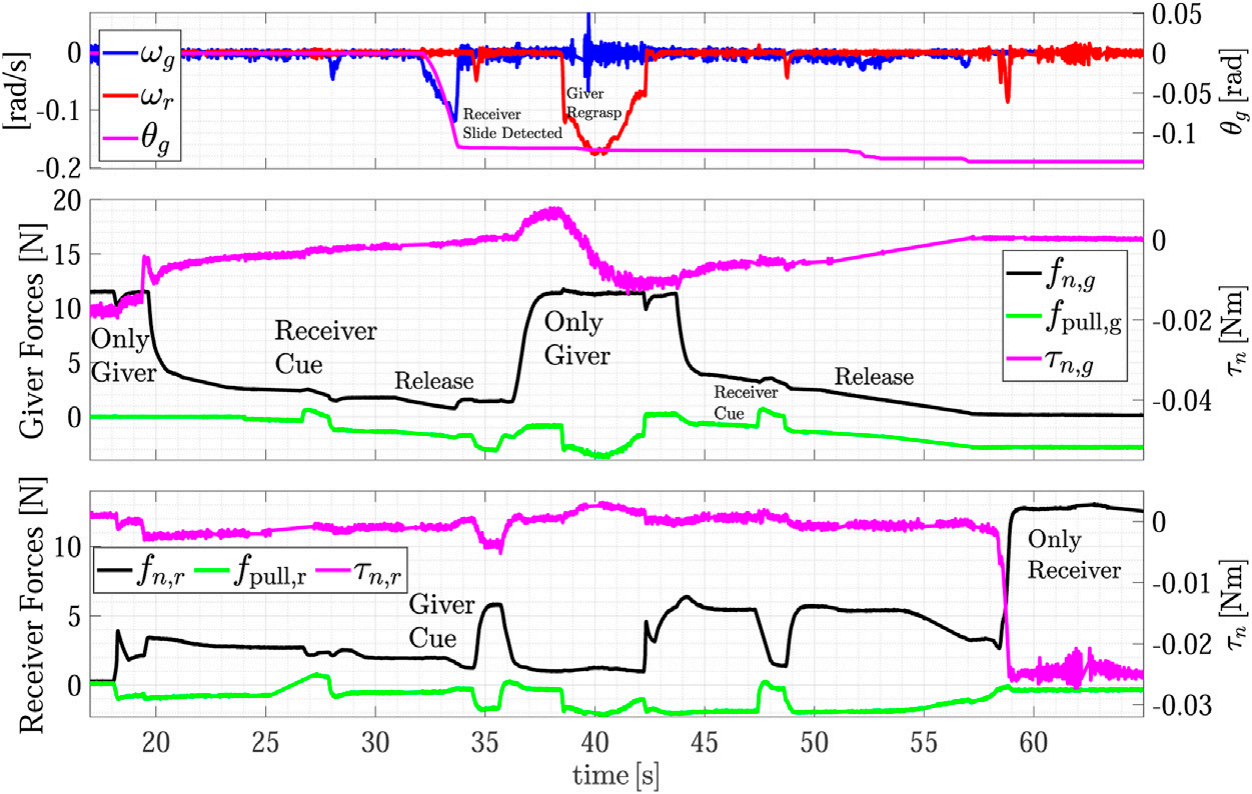
Fourth experiment (Object C): estimated slipping velocities of the giver and the receiver **(top);** forces and torque measured by the giver **(middle plot);** forces and torque measured by the receiver **(bottom plot)**.

The R2R handover experiment has been carried out with Object C. Figure 13 shows the snapshots of the experiment execution, while the forces and torque as well as the estimated slipping velocities of the giver $\left(\omega_{g}\right)$ and of the receiver $\left(\omega_{r}\right)$ are reported in Figure 14. At *t* = 0 s the SIA5F grasps the object in a given position and lifts it in SA mode. The grasp controller applies a force of 11 N that is needed to counteract the gravitational torque even if the object weight force is less than 1 N. Then, the giver presents the object to the receiver switching to the *Wait* state. The receiver starts moving toward it guided by the visual servoing controller (*t* = 13 s). At *t* = 18 s the receiver starts grasping the object and the physical handover begins. When the receiver completes the grasp, the torsional load $\tau_{n,g}$ on the giver decreases, this, in turn, causes a reduction of the giver grasp force $f_{n,g}$ (*t* = 20 s). At *t* = 27 s the receiver decides to give the haptic cue to the giver, which detects the event (Eq. 17) by checking condition (Eq. 17). This is evident in the middle plot of Figure 14 where $f_{\text{pull},g}$ overcomes the threshold $\psi_{p}$. Then, the giver enters the release state and starts releasing the object (*t* = 30 s). During this phase (at *t* = 32 s), the grasp force declines such that the giver foresees a virtual slipping by detecting the condition (Eq. 18) since the virtual sliding angle $\left|\vartheta_{g}\right|$ overcomes the threshold $\psi_{\theta }=0.087\;\text{rad}$. This way, the giver robot is able to foresee a slipping event that would happen if it continued the grip force decreasing to pass the object load to the receiver, and this without any communication between the robots. Therefore, the giver aborts the opening and comes back to slipping avoidance mode. It sends an haptic cue to the receiver communicating that it cannot support the object. The receiver detects such event with the condition (Eq. 19) and, as stated in Section 3.3, it moves toward the giver to grasp the object closer to its center of gravity by a displacement of 2.5 cm (Regrasp state). Assuming the parallelepiped shape, the giver can move by applying a low normal force of 1 N to avoid contact loss, and by letting the fingers slide on the object surface. This is shown also in the top plot of Figure 14 from *t* = 38 to *t* = 42 s when the observer on the receiver estimates a sliding velocity $\omega_{r}$, which corresponds to the translational sliding motion.

After the sliding phase, the receiver goes back to SA mode and at *t* = 47 s, it sends again the cue to the giver to grasp the object. This time, during the release phase from *t* = 50 to *t* = 55 s the variation on the virtual sliding angle estimated by the giver $\vartheta_{g}$ does not overcame the threshold $\psi_{\vartheta }$ and the giver completely releases the object. From the receiver point of view, the grasping force computed by the SA is mainly due to the pulling force $f_{{\text{pull}}_{r}}$ since the object weight is very small and the torsional moment $\tau_{n,r}$ is negligible. Therefore, the lower the pulling force the lower the grip force $f_{n,r}$, until the receiver feels a significant increment of the external torque $\tau_{n,r}$ (*t* = 58 s) and a peak in the estimated velocity $\omega_{r}$. The velocity peak notifies the receiver FSM that the handover task is complete [condition (Eq. 20)], while both the velocity peak and the torque increment are used by the SA algorithm to increase the grasp force up to 12.5 N.

## 5 Conclusion

The experimental results reported in the paper give evidence to the importance of the haptic perception during the handover operations between humans and robots but even between robots that communicate only through physical interaction. The force/tactile perception enables reactive controllers to smartly modulate the grip force during the physical handover phase, ensuring successful handovers without object slippage. The proposed methods, based on physics models of the soft contact, have been presented in the framework of an in-store logistic collaborative scenario with a set of requirements and assumptions, and, in particular, using parallel grippers. Nevertheless, satisfactory results encourage us to investigate possible generalization to more complex robotic grippers, which can enlarge the set of objects that can be handled. We presented not only classical experiments of human-to-robot handovers and vice versa, but also a preliminary algorithm for robot-to-robot handover, that is envisaged useful in a future where robots collaborate with each other with simple communication channels, like haptic cues. The current limitation is the assumption of objects with specific shapes and the knowledge of the location of the re-grasp point. Overcoming this limit requires methods to re-plan or learn the re-grasping strategy.

## Data Availability

The original contributions presented in the study are included in the article/Supplementary Material, further inquiries can be directed to the corresponding author.

## Footnotes

1 World Robotic Summit (2021). Future Convenience Store Challenge. Available at: https://worldrobotsummit.org/en/wrs2020/challenge/service/fcsc.html (Accessed April 20, 2021).

2 Sameer Agarwal and Keir Mierle and Others (2021). Ceres Solver. Available at: http://ceres-solver.org (Accessed April 20, 2021).

## Supplementary Material

SUPPLEMENTARY VIDEO S1 | The experiments carried out to demonstrate the R2H, H2R, and R2R handover strategies.

## References

[B1] Aleotti J. Micelli V. Caselli S. (2014). An Affordance Sensitive System for Robot to Human Object Handover. Int. J. Soc. Robotics 6, 653–666. 10.1007/s12369-014-0241-3

[B2] Bogue R. (2019). Strong Prospects for Robots in Retail. Ir 46, 326–331. 10.1108/ir-01-2019-0023

[B3] Canudas de Wit C. Olsson H. Aström K. J. Lischinsky P. (1995). A New Model for Control of Systems with Friction. IEEE Trans. Automat. Contr. 40, 419–425. 10.1109/9.376053

[B4] Cavallo A. Costanzo M. De Maria G. Natale C. (2020). Modeling and Slipping Control of a Planar Slider. Automatica 115, 108875. 10.1016/j.automatica.2020.108875

[B5] Chan W. P. Parker C. A. C. Van der Loos H. F. M. Croft E. A. (2012). “Grip Forces and Load Forces in Handovers: Implications for Designing Human-Robot Handover Controllers,” in 2012 7th ACM/IEEE International Conference on Human-Robot Interaction (HRI), Boston, MA, March 5–8, 2012, 9–16. 10.1145/2157689.2157692

[B6] Costanzo M. De Maria G. Lettera G. Natale C. (2021). Can Robots Refill a Supermarket Shelf?: Motion Planning and Grasp Control. IEEE Robot. Automat. Mag. 2021, 2–14. 10.1109/MRA.2021.3064754

[B7] Costanzo M. De Maria G. Lettera G. Natale C. (2020a). Grasp Control for Enhancing Dexterity of Parallel Grippers,” in 2020 IEEE International Conference on Robotics and Automation, Paris, France, May 31–August 31, 2020, 524–530.

[B8] Costanzo M. De Maria G. Natale C. Pirozzi S. (2019). Design and Calibration of a Force/tactile Sensor for Dexterous Manipulation. Sensors 19, 966. 10.3390/s19040966 PMC6412380 30823548

[B9] Costanzo M. De Maria G. Natale C. (2020b). Two-fingered In-Hand Object Handling Based on Force/tactile Feedback. IEEE Trans. Robot. 36, 157–173. 10.1109/tro.2019.2944130

[B10] Costanzo M. (2020). Soft-contact Modeling For In-Hand Manipulation Control and Planning. PhD thesis. Aversa, Italy: Università degli Studi della Campania Luigi Vanvitelli

[B11] Costanzo M. Stelter S. Natale C. Pirozzi S. Bartels G. Maldonado A. (2020c). Manipulation Planning and Control for Shelf Replenishment. IEEE Robot. Autom. Lett. 5, 1595–1601. 10.1109/LRA.2020.2969179

[B12] Endo S. Pegman G. Burgin M. Toumi T. Wing A. M. (2012). “Haptics in Between-Person Object Transfer,” in Haptics: Perception, Devices, Mobility, and Communication. Berlin Heidelberg: Springer, 103–111. 10.1007/978-3-642-31401-8_10

[B13] Howe R. D. Cutkosky M. R. (1996). Practical Force-Motion Models for Sliding Manipulation. Int. J. Robotics Res. 15, 557–572. 10.1177/027836499601500603

[B14] Kuhn H. Sternbeck M. G. (2013). Integrative Retail Logistics: An Exploratory Study. Oper. Manag. Res. 6, 2–18. 10.1007/s12063-012-0075-9

[B15] Marchand E. Spindler F. Chaumette F. (2005). ViSP for Visual Servoing: a Generic Software Platform With a Wide Class of Robot Control Skills. IEEE Robot. Automat. Mag. 12, 40–52. 10.1109/mra.2005.1577023

[B16] Mason A. H. MacKenzie C. L. (2005). Grip Forces when Passing an Object to a Partner. Exp. Brain Res. 163, 173–187. 10.1007/s00221-004-2157-x 15761722

[B17] Medina J. R. Duvallet F. Karnam M. Billard A. (2016). “A Human-Inspired Controller for Fluid Human-Robot Handovers,” in 2016 IEEE-RAS 16th International Conference on Humanoid Robots (Humanoids), Cancun, Mexico, November 15–17, 2016, 324–331. 10.1109/HUMANOIDS.2016.7803296

[B18] Nemlekar H. Dutia D. Li Z. (2019). Object Transfer Point Estimation for Fluent Human-Robot Handovers,” in 2019 International Conference on Robotics and Automation, Montréal, QC, May 20–24, 2019 (Montréal, QC: ICRA), 2627–2633. 10.1109/ICRA.2019.8794008

[B19] Ortenzi V. Cosgun A. Pardi T. Chan W. Croft E. Kulic D. (2020). Object Handovers: a Review for Robotics. arXiv:2007.12952

[B20] Pan M. K. X. J. Croft E. A. Niemeyer G. (2018). “Exploration of Geometry and Forces Occurring within Human-To-Robot Handovers,” in 2018 IEEE Haptics Symposium (HAPTICS), San Francisco, CA, March 25–28, 2018, 327–333. 10.1109/HAPTICS.2018.8357196

[B21] Retail Analytics Council (2020). Emerging Trands in Retail Robotics. Evanston, IL: Northwestern University, 1–24.

[B22] Ricardez G. A. G. Okada S. Koganti N. Yasuda A. Eljuri P. M. U. Sano T. (2020). Restock and Straightening System for Retail Automation Using Compliant and Mobile Manipulation. Adv. Robotics 34, 235–249. 10.1080/01691864.2019.1698460

[B23] Rosenberger P. Cosgun A. Newbury R. Kwan J. Ortenzi V. Corke P. (2021). Object-independent Human-To-Robot Handovers Using Real Time Robotic Vision. IEEE Robot. Autom. Lett. 6, 17–23. 10.1109/LRA.2020.3026970

[B24] Sakai R. Katsumata S. Miki T. Yano T. Wei W. Okadome Y. (2020). A Mobile Dual-Arm Manipulation Robot System for Stocking and Disposing of Items in a Convenience Store by Using Universal Vacuum Grippers for Grasping Items. Adv. Robotics 34, 219–234. 10.1080/01691864.2019.1705909

[B25] Sartori L. Straulino E. Castiello U. (2011). How Objects Are Grasped: the Interplay between Affordances and End-Goals. PLoS One 6, e25203. 10.1371/journal.pone.0025203 21980396 PMC3182194

[B26] Shkulipa S. A. den Otter W. K. Briels W. J. (2005). Surface Viscosity, Diffusion, and Intermonolayer Friction: Simulating Sheared Amphiphilic Bilayers. Biophysical J. 89, 823–829. 10.1529/biophysj.105.062653 PMC1366632 15894643

[B27] Strabala K. W. Lee M. K. Dragan A. D. Forlizzi J. L. Srinivasa S. Cakmak M. (2013). Towards Seamless Human-Robot Handovers. Jhri 2, 112–132. 10.5898/JHRI.2.1.Strabala

[B28] Xydas N. Kao I. (1999). Modeling of Contact Mechanics and Friction Limit Surfaces for Soft Fingers in Robotics, with Experimental Results. Int. J. Robotics Res. 18, 941–950. 10.1177/02783649922066673

[B29] Yang W. Paxton C. Cakmak M. Fox D. (2020). Human Grasp Classification for Reactive Human-To-Robot Handovers,” in 2020 IEEE/RSJ International Conference on Intelligent Robots and Systems (IROS), Las Vegas, NV, October 24, 2020, 11123–11130. 10.1109/IROS45743.2020.9341004

